# Long Noncoding RNA *lnc-TSSK2-8* Activates Canonical Wnt/β-Catenin Signaling Through Small Heat Shock Proteins HSPA6 and CRYAB

**DOI:** 10.3389/fcell.2021.660576

**Published:** 2021-05-10

**Authors:** Jingjing Fa, Xiaoqing Zhang, Xiaoping Zhang, Ming Qi, Xingyu Zhang, Qihua Fu, Zhuoming Xu, Yunqian Gao, Bo Wang

**Affiliations:** ^1^Pediatric Translational Medicine Institute, Shanghai Children’s Medical Center, Shanghai Jiao Tong University School of Medicine, Shanghai, China; ^2^Department of Laboratory Medicine, Shanghai Children’s Medical Center, Shanghai Jiao Tong University School of Medicine, Shanghai, China; ^3^Faculty of Medical Science, Shanghai Jiao Tong University School of Medicine, Shanghai, China; ^4^Cardiac Intensive Care Unit, Department of Thoracic and Cardiovascular Surgery, Shanghai Children’s Medical Center, Shanghai Jiao Tong University School of Medicine, Shanghai, China

**Keywords:** congenital heart disease, long noncoding RNA, 22q11.2 deletion, *lnc-TSSK2-8*, Wnt/β-catenin signaling

## Abstract

Congenital heart defects (CHDs) are the most common birth defects worldwide. 22q11.2 deletion syndrome is the most common microdeletion disorder that has been frequently associated with conotruncal malformations. By now, the dosage-sensitive gene *TBX1* has been adopted as the major pathogenic gene responsible for 22q11.2 deletion, which is regulated by canonical Wnt/β-catenin signaling pathway in heart outflow tract development. Here, we report the long noncoding RNA (lncRNA) *lnc-TSSK2-8*, which is encompassed in the 22q11.2 region, that can activate canonical Wnt/β-catenin signaling by protecting β-catenin from degradation, which could result from decreased ubiquitination. Such effects were mediated by two short heat shock proteins HSPA6 and α-β-crystallin (CRYAB), whose expression was regulated by *lnc-TSSK2-8* through a competing endogenous RNA (ceRNA) mechanism. In clinical practice, the pathogenesis of copy number variation (CNV) was always attributed to haploinsufficiency of protein-coding genes. Here, we report that the 22q11.2 lncRNA *lnc-TSSK2-8* significantly activated canonical Wnt/β-catenin signaling, which has major roles in cardiac outflow tract development and should act upstream of *TBX1*. Our results suggested that lncRNAs should contribute to the etiology of CNV-related CHD.

## Introduction

Congenital heart defects (CHDs) are the most common birth defects worldwide, with an incidence of nearly 10∼12 per 1,000 live infants (1∼1.2%) (Hoffman and Kaplan, 2002; Van Der Linde et al., 2011). As a consequence of improvement in surgical and medical management, more and more patients with severe forms of CHDs can survive until their 30s and beyond. Genetic variation, environmental causes, and their interactions could contribute to the disruption of heart development, thus leading to formation of CHDs (Pierpont et al., 2018; Zu et al., 2020). Genetic variations associated with CHDs have been revealed extensively in recent years. Single gene disorder and gross chromosomal anomalies/aneuploidy can be identified in up to 5 and 10% CHD cases, respectively. Copy number variations (CNVs) are segmental deletions or duplications ranging widely in size of millions of base pairs; pathogenic CNVs could explain about 3∼25% syndromic CHD and 3∼10% isolated CHD cases (Cowan and Ware, 2015).

22q11.2 deletion syndrome (22q11.2DS) is the most common microdeletion disorder with an estimated prevalence ranging from 1 per 3,000 to 1 per 6,000 live births (Tezenas Du Montcel et al., 1996; Devriendt et al., 1998; Goodship et al., 1998; Botto et al., 2003; Oskarsdottir et al., 2004). It is well documented that 22q11.2DS involves 0.7∼3 million base pairs, resulting in multiorgan dysfunction including CHDs, palatal abnormalities, immunodeficiency, developmental delays, cognitive deficits, and neuropsychiatric illnesses. 22q11.2 deletion is the second most common cause of CHDs; conotruncal malformations [malformations of the outflow tract (OFT) such as the tetralogy of Fallot (TOF), truncus arteriosus, interrupted aortic arch type B, and ventricular septal defect (VSD)] account for ∼70% of the heart defects associated this CNV (Agergaard et al., 2012). On the other hand, CHD is also the main cause of mortality (∼87%) in 22q11.2DS patients (McDonald-McGinn et al., 2001; Repetto et al., 2014).

Since the function of proteins had been well annotated, most clinical pathogenic CNVs were interpreted based on their effect on gene dosage of protein-coding genes. There are 46 protein-coding genes located in the typical 3M region of 22q11.2 locus, among which most studies of interest relate to *TBX1*, which encodes a T-box transcription factor. *TBX1* was found to be a crucial gene in the LCR22A–LCR22B region; heterozygous loss of function mutations of *Tbx1* in the mouse resulted in penetrant defects that are reminiscent of CHD in 22q11.2 (Lindsay et al., 2001; Chapnik et al., 2012). Another gene of interest is *DGCR8*, which encodes a double-stranded RNA-binding protein that is involved in microRNA (miRNA) biogenesis. Heterozygosity of *Dgcr8* results in neuronal deficits (Stark et al., 2008), whereas ablation of both alleles in neural crest cells causes heart defects (Chapnik et al., 2012). Additionally, evidence of other individual protein-coding genes in 22q11.2 region responsible for major cardiac phenotypes of 22q11.2DS has also accumulated. For example, haploinsufficiency of *CRKL* could account for the cardiac anomalies in individuals with nested distal deletions (Zheng et al., 2015).

Long noncoding RNAs (lncRNAs), which contain >200 nucleotides, have been revealed to participate in the regulation of cellular and tissue function and play a great part in heart development (Devaux et al., 2015). LncRNAs can repress or activate gene expression epigenetically with diverse mechanisms: e.g., most lncRNA molecules localize in the nucleus and regulate gene expression epigenetically; a proportion (∼15%) of lncRNA are located in the cytoplasm where they function posttranscriptionally (Devaux et al., 2015). Several lncRNAs have been identified as key regulatory molecules involved in cardiac development, such as *Braveheart* (Klattenhoff et al., 2013), *Fendrr* (Grote and Herrmann, 2013), *Upperhand* (Anderson et al., 2016), and *BANCR* (Wilson et al., 2020). Several examples of CHD-associated lncRNAs have also been reported. Gu et al. (2016) indicated that circulating plasma lncRNAs might serve as novel biomarkers for CHD. Jiang et al. (2018) discovered that *HOTAIR* is upregulated in cardiac tissues and plasma of patients with CHD; thus, it could serve as a potential diagnostic biomarker for CHD. Wang et al. (2018) suggested the relationship between *HA117* and TOF, although the molecular basis of *HA117* remains unclear. Jiang et al. (2019) reported upregulated *SNHG6* expression in fetal heart tissues of patients with VSDs. Ma et al. (2020) reported that *TBX5-AS1* should be involved in TOF by affecting cell proliferation by regulating TBX5. This evidence signifies the importance of lncRNA in CHD. Haploinsufficiency of *TBX1* is regarded as the major candidate for cardiac OFT malformation. Recently, canonical Wnt/β-catenin has been revealed to play major roles in cardiac OFT development upstream of *TBX1* (Racedo et al., 2017). It is unknown whether lncRNAs participate in this process. The canonical Wnt/β-catenin signaling pathway has been revealed to play developmental stage-specific roles in early heart development. The Wnt family proteins serve as secreted signaling molecules that initiate a series of intracellular pathways. In the canonical Wnt/β-catenin signaling pathway, Wnt signaling starts with the generation of Wnt ligands into the extracellular space. The Wnt molecules then bind to the Frizzled receptor (Frz) and its coreceptor LRP5/6 to form trimeric complexes. The formation of such complexes inhibits the glycogen synthase kinase 3β (GSK-3β) destruction complex, which functions in stabilizing the cytosolic β-catenin (Blankesteijn et al., 2008). Cytosolic accumulation of β-catenin leads to its translocation into the nucleus. Subsequently, the Wnt proteins bind to the T-cell factor (TCF)/lymphoid enhancer-binding factor (LEF) transcription factors to activate Wnt-responsive genes. Activation of Wnt/β-catenin signaling during embryoid body (EB) formation promotes embryonic stem cell differentiation into cardiomyocytes. Whereas the activation of Wnt/β-catenin signaling in a later stage (during gastrulation) results in inhibition of cardiomyocyte formation (Naito et al., 2006; Ueno et al., 2007).

In the present study, we discovered that the lncRNA *lnc-TSSK2-8*, which is located in the 22q11.2 region, significantly promotes the stabilization of β-catenin. Therefore, we propose that *lnc-TSSK2-8* should contribute to heart development by regulating Wnt/β-catenin signaling pathway. In contrast to earlier studies that emphasize the crucial roles of protein-coding genes, these data suggest that the relationship between disease and noncoding transcripts should be further explored.

## Materials and Methods

### Rapid Amplification of Complementary DNA Ends

Here, 5′ and 3′ rapid amplification of complementary DNA (cDNA) ends (RACE) was performed on RNA isolated from HEK293 cells using a SMARTer RACE 5′/3′ Kit (TAKARA, Dalian, China), following the manufacturer’s instructions. The PCR products were separated on a 1% agarose gel and validated by Sanger sequencing. The following gene-specific primers were used for PCR:

GACTGAAGGAGTAGAAA (5′ RACE primer)
GTCCAGGTGTCCCTGCCTCCCATTG (3′ RACE primer 1)
GGGGGAAGCCCACAATGAGCAG (3′ RACE primer 2)

### Isolation of Nuclear and Cytoplasmic RNA

Nuclear RNA and cytoplasmic RNA of HEK293 cells were extracted and purified using the PARIS^TM^ Kit (Invitrogen, Thermo Fisher Scientific, Inc., Waltham, MA, United States) according to the manufacturer’s instructions.

### Cell Culture and Long Noncoding RNA Overexpression

The HEK293 cell line was purchased from the Type Culture Collection of the Chinese Academy of Sciences, Shanghai, China. Cells were cultured with Dulbecco’s modified Eagle’s medium (DMEM; Gibco, Thermo Fisher Scientific, Inc., Waltham, MA, United States) containing 10% fetal bovine serum (FBS; Gibco, Thermo Fisher Scientific, Inc., Waltham, MA, United States) and 0.1% penicillin/streptomycin (NCM Biotech, Suzhou, Jiangsu, China) at 37°C with 5% CO_2_.

The cDNA sequence of *lnc-TSSK2-8* was inserted into pcDNA3.1 (pcDNA3.1-lncTSSK2.8) for overexpression. HEK293 cells were transfected with pcDNA3.1-lncTSSK2.8 by using Lipofectamine 3000 (Invitrogen, Thermo Fisher Scientific, Inc., Waltham, MA, United States) according to the manufacturer’s instruction.

### RNA Sequencing Analysis

Cell samples were lysed in TRIzol (Ambion, Austin, TX, United States). Total RNA was extracted using the RNeasy kit (Qiagen, Hilden, Germany) according to the manufacturer’s instructions.

A total amount of 2 μg RNA per sample was used as input material for the RNA preparations. Firstly, ribosomal RNA was removed using Epicenter Ribo-zero^TM^ Kit (Illumina Inc., San Diego, CA, United States), and rRNA-free residue was cleaned up by ethanol precipitation. By using the fragmentation buffer, the mRNA and noncoding RNAs were fragmented into short fragments (about 200∼700 bp), then the first-strand cDNA was synthesized by random hexamer-primer using the fragments as templates. Buffer, dNTPs, RNase H, and DNA polymerase I were added to synthesize the second strand cDNA. Subsequently, A-Tailing Mix (QIAGEN, Hilden, Germany) and RNA Index Adapters were added to perform end-repair, and the resulting cDNA was amplified by PCR. The double-strand cDNA was purified with QiaQuick PCR extraction kit and then used for end-polishing. Sequencing adapters were ligated to the fragments, then the second strand was degraded using Uracil-N-Glyosylase (UNG) finally. The quantity and quality of cDNA libraries were evaluated using Agilent 2100 Bioanalyzer (ABI, New York, NY, United States). The double-stranded PCR products were heat denatured and circularized by the splint oligo sequence to obtain the final library. The single-stranded circular DNA was amplified using phi29 (Thermo Fisher Scientific, Waltham, MA, United States) to generate a DNA nanoball. The library preparations were sequenced on BGISEQ-500 platform, and 150-bp paired-end reads were generated.

Raw sequencing reads with low quality (e.g., proportion of bases with sQ ≤ 5 greater than 50%; proportion of N greater >10%; 5′adaptor contamination) were removed using customized scripts. The remaining clean data were aligned to the human reference genome (version GRCh38) using STAR (Dobin et al., 2013) with standard options for long RNA sequencing (RNAseq) pipeline of Encyclopedia of DNA Elements (ENCODE;^1^): –outFilterType BySJout, –outFilterMultimapNmax 20, –alignSJoverhangMin 8, –alignSJDBoverhangMin1, –outFilterMismatchNmax 999, –outFilterMismatchNoverReadLmax 0.04, –alignIntronMin 20, –alignIntronMax 1000000, –alignMatesGapMax 1000000. Gene level expression was estimated using the featureCounts software (Liao et al., 2014). Differential gene expression analysis was performed on the raw counts using the DESeq2 (Love et al., 2014) package. Genes with fold change greater than 2 and *P* value lower than 0.05 were identified as differentially expressed genes (DEGs). Functional enrichment analysis was performed with the clusterProfiler (Yu et al., 2012) package; gene set enrichment analysis was performed for Gene Ontology (GO) items [Biological Process (BP), Molecular Function (MF), Cell Component (CC)] and Kyoto Encyclopedia of Genes and Genomes (KEGG) pathway items. We used the genes with fold change greater than 1.2 for functional enrichment analysis, since the DEG number is too small for such analysis. BH adjustments were used for multiple testing of functional enrichment analysis; *p* < 0.05 and *q* < 0.05 were used as thresholds.

### RT-qPCR

Total RNA was extracted with TRIzol reagent (Ambion, Austin, TX, United States) and reverse transcribed into cDNA with PrimeScript RT reagent Kit (Takara, Dalian, China). RT-qPCR was conducted with TB Green Premix Ex Taq II kit (Takara, Dalian, China) on the CFX 9600 Real-Time PCR detection system (Bio-Rad Laboratories, Inc., Hercules, CA, United States). Fold changes of the lncRNA and mRNAs were calculated with small nuclear RNA U6 and *glyceraldehyde 3-phosphate dehydrogenase (GAPDH)* as internal controls, respectively, based on 2^–Δ^ ^Δ^ ^*Ct*^ method. All the Bulge-Loop RT primers for both microRNAs and U6 were purchased from RiboBio (Guangzhou, Guangdong, China).

### Luciferase Reporter Assay

*Lnc-TSSK2-8* wild type/mutation (WT/MUT) and heat shock protein (HSP)A6 UTR WT/MUT were inserted into the BamH I and Hind III sites of the pMIR dual-luciferase vector (Promega, Madison, WI, United States). These plasmids as well as the pMIR vector were transfected with the miR-6721-5p mimics or NC-mimics into HEK293 cells for 48 h, respectively. Finally, luciferase activities were detected using the Dual-Luciferase Reporter Assay System (Promega, Madison, WI, United States) according to the manufacturer’s protocol.

### Western Blotting

The transfected cells were disintegrated using Western blot (WB) IP lysis buffer (Beyotime Biotechnology, Shanghai, China). All protein extracts were separated with 10% sodium dodecyl sulfate–polyacrylamide gel electrophoresis (SDS-PAGE; Beyotime, Nantong, Jiangsu, China), transferred onto polyvinylidene fluoride (PVDF) membranes (Roche Diagnostic Corporation, Indianapolis, IN, United States) and blotted with primary antibodies (HSPA6, Abcam, Cat. No. ab69408; β-Catenin, Cell Signaling Technology, Cat. No. 8480S; p-β-Catenin, Cell Signaling Technology, Cat. No. 5651S; GAPDH, Cell Signaling Technology, Cat. No. 5174S; Tubulin, Cell Signaling Technology, Cat. No. 5335S). Sequentially, membranes were incubated with secondary antibodies (1:10,000; Cell Signaling Technology, Cat. No. 7074). At last, proteins were visualized using ECL detection kits (Millipore Corp., Millipore Billerica, MA, United States).

### BrdU Cell Proliferation Assay

The cell proliferation assay was performed using TransDetect EdU Flow Cytometry Kit-647 Fluorophore (TransGen Biotech, Beijing, China). Cells were cultured with EdU solution for 2 h and fixed by 1× EdU permeabilization buffer. The assay was visualized by the FACScan system (Bio-Rad Laboratories, Inc., Hercules, CA, United States).

### Statistical Analysis

The SPSS 16.0 software was used for statistical analysis. Differences between two groups were analyzed by implementing Student *t*-test, and *P*-value < 0.05 was regarded as statistically significant. All data were expressed as mean ± SD.

## Results

### Full-Length Characterization and Subcellular Localization of *lnc-TSSK2-8*

**FIGURE 1:**
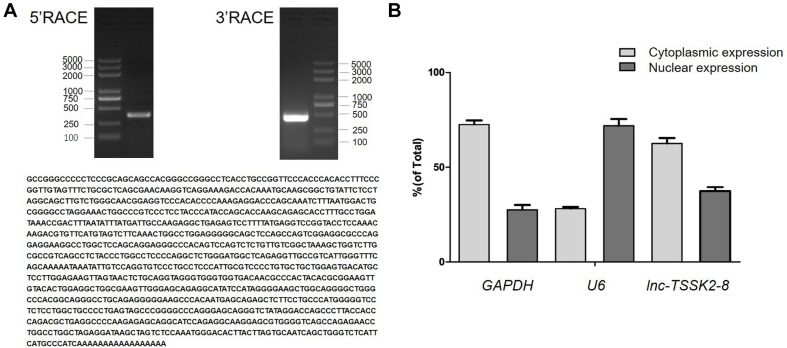
Full-length characterization and subcellular expression analysis of *lnc-TSSK2-8*. **(A)** Electrophoresis analysis of rapid amplification of cDNA ends (RACE) PCR and the obtained full-length cDNA sequence of *lnc-TSSK2-8*. **(B)** Subcellular distribution of *lnc-TSSK2-8*. Glyceraldehyde 3-phosphate dehydrogenase (GAPDH) and U6 were used for references of cytoplasmic expression and nuclear expression, respectively.

The full-length sequence of *lnc-TSSK2-8* was successfully obtained from HEK293 cells by rapid amplification of the 5′ and 3′ cDNA assays. We confirmed the sequence by performing PCR and Sanger sequencing. The full-length transcript of *lnc-TSSK2-8* was 1,128 in size with a polyadenylated tail (Figure 1A). To determine the subcellular localization of *lnc-TSSK2-8*, we separated the nuclear and cytoplasm fractions of HEK293 cells and quantified its expression with RT-qPCR. The results suggested that *lnc-TSSK2-8* is located both in the nucleus and cytoplasm, although the cytoplasmic composition was much more abundant (Figure 1B).

### *lnc-TSSK2-8* Activates Canonical Wnt/β-Catenin Signaling

**FIGURE 2:**
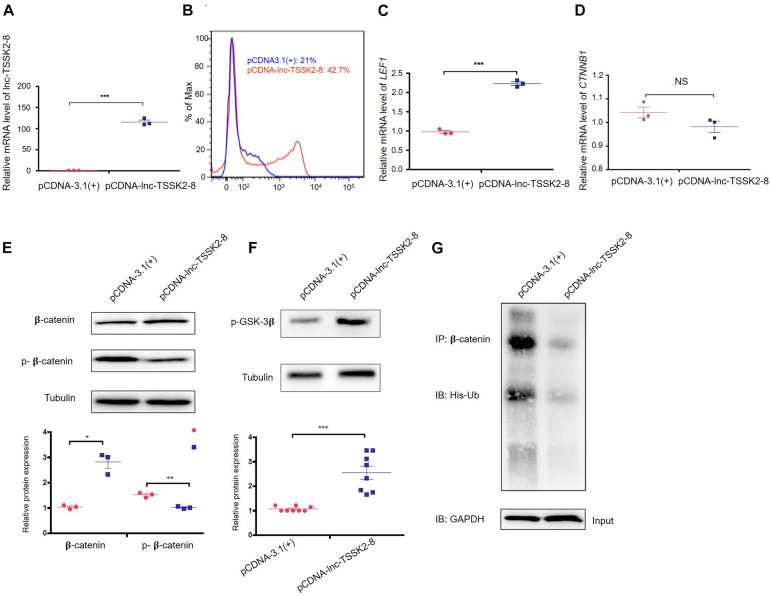
Overexpression of *lnc-TSSK2-8* activates the Wnt/β-catenin signaling pathway. **(A)** RT-qPCR quantification of overexpression *lnc-TSSK2-8* in HEK293 cells. **(B)** Flow cytometry analysis of cell proliferation. The transcriptional level of lymphoid enhancer-binding factor 1 (*LEF1*) **(C)** and β-catenin (*CTNNB1*) **(D)** in HEK293 cells with *lnc-TSSK2-8* overexpression. **(E)** The influence of *lnc-TSSK2-8* overexpression on protein expression and phosphorylation of β-catenin. **(F)** The influence of *lnc-TSSK2-8* overexpression on the activity of glycogen synthase kinase 3β (GSK-3β). **(G)** The influence of *lnc-TSSK2-8* overexpression on ubiquitination of β-catenin. *p < 0.05, **p < 0.001, **p < 0.0001.

We overexpressed the full-length transcript of *lnc-TSSK2-8* in HEK293 cells (Figure 2A) and examined the expression of the key components of canonical Wnt/β-catenin signaling pathway. Overexpression of *lnc-TSSK2-8* increased cell proliferation by ∼20% (Figure 2B) and activated the transcription of the LEF (*LEF1*) (Figure 2C), whereas the transcriptional level of β-catenin (CTNNB1) did not change (Figure 2D). Further exploration suggested that the protein expression level of β-catenin increased significantly; such an increase should be associated with its decreased phosphorylation (Figures 2E,G). Subsequently, we revealed that the phosphorylation of GSK-3β (Figure 2F) was enhanced and established that ubiquitination of β-catenin could be inhibited by *lnc-TSSK2-8.*

### Transcriptomic Analysis of *lnc-TSSK2-8* Overexpression

**FIGURE 3:**
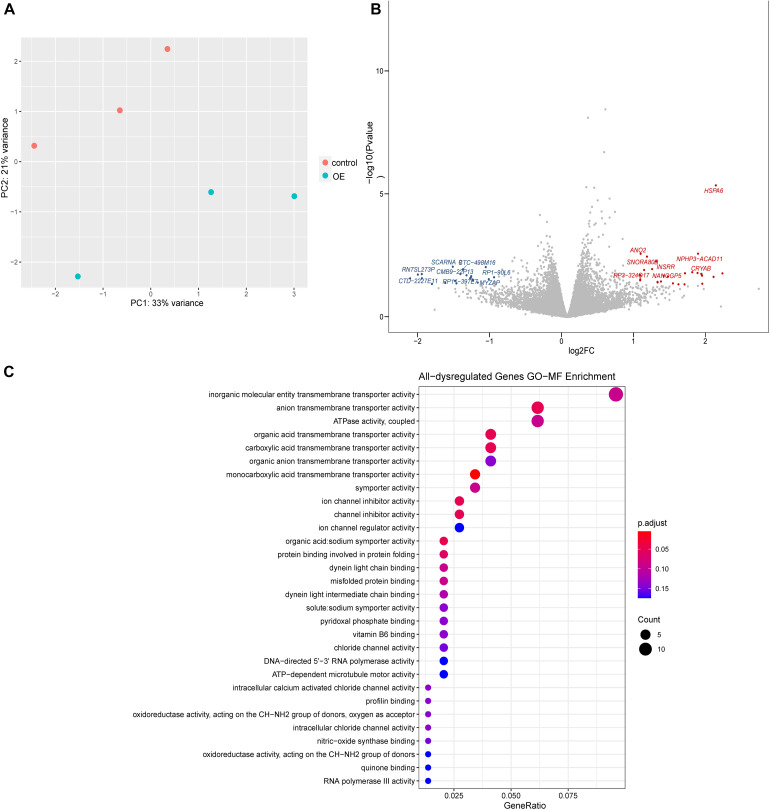
Differential gene expression analysis of *lnc-TSSK2-8* overexpression in HEK293 cells. **(A)** Principal component analysis of RNAseq data derived from *lnc-TSSK2-8* overexpression and control cell samples. **(B)** Volcano plot of differential gene expression analysis; symbols of the top 15 for each of upregulated and downregulated genes were labeled. **(C)** Gene Ontology (GO) gene set functional enrichment analysis of DEGs.

To explore the molecular basis of Wnt/β-catenin signaling regulation, we performed transcriptomic analysis. Our results indicated that overexpression of *lnc-TSSK2-8* resulted in differential expression of 62 genes/lncRNAs, among which *HSPA6* was the most significantly upregulated (*p* = 1.27 × 10^–5^) (Figures 3A,B). GO gene set enrichment analysis indicated that the DEGs were enriched in MFs such as transmembrane transporter activities (Figure 3C). KEGG enrichment analysis indicated that the DEGs were enriched in beta-Alanine metabolism (*p* < 0.05).

### *lnc-TSSK2-8* Activates Wnt/β-Catenin Signaling Through a Competing Endogenous RNA Mechanism

**FIGURE 4:**
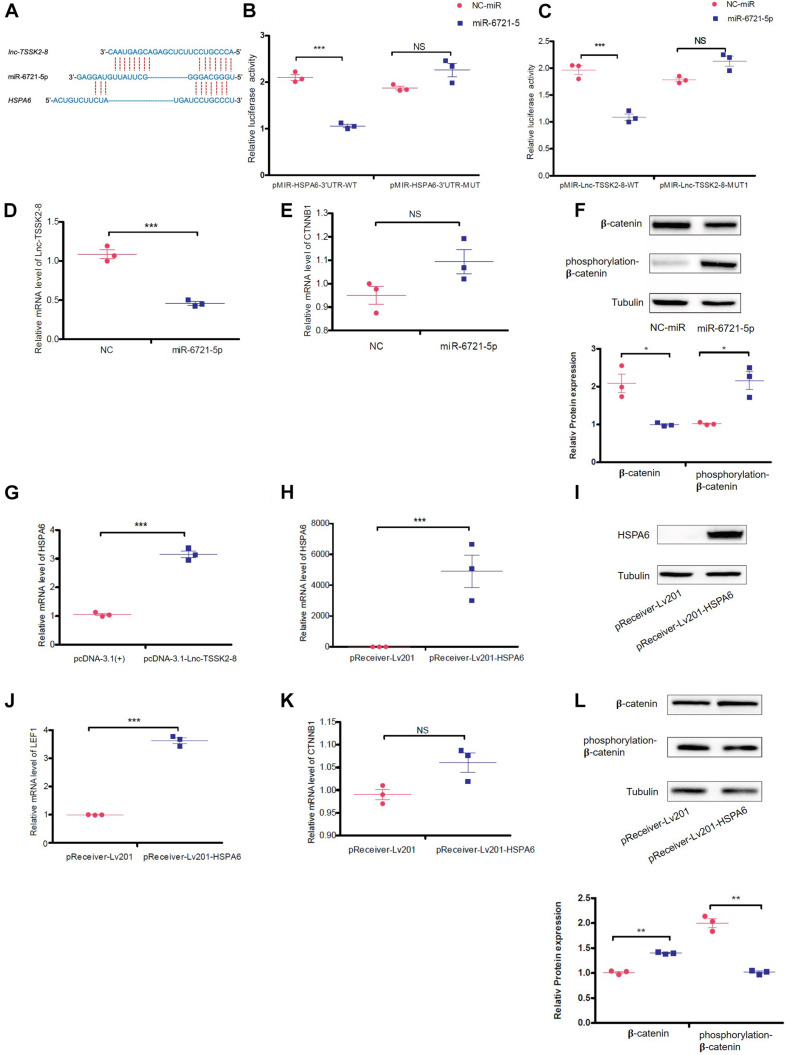
*lnc-TSSK2-8* activation promotes β-catenin stabilization through miR-6721-5p/heat shock protein (HSP)A6 axis. **(A)** The predicted miR-6721-5p binding sites of *lnc-TSSK2-8* and *HSPA6* 3′ UTR. **(B)** The luciferase reporter assay we performed to confirm the interaction between miR-6721-5p and *HSPA6* 3′ UTR **(B)**/*lnc-TSSK2-8* **(C)**. **(D)** RT-qPCR analysis of *lnc-TSSK2-8* level in response to miR-6721-5p overexpression. **(E)** RT-qPCR analysis of β-catenin level in response to miR-6721-5p overexpression. **(F)** Western blot (WB) analysis of protein expression and phosphorylation of β-catenin in response to miR-6721-5p overexpression. **(G)** RT-qPCR analysis of *HSPA6* level in response to *lnc-TSSK2-8* overexpression. **(H)** Confirmation of *HSPA6* overexpression with RT-qPCR. **(I)** Confirmation of HSPA6 overexpression with WB. **(J)** RT-qPCR analysis of *LEF1* level in response to *HSPA6* overexpression. **(K)** RT-qPCR analysis of β-catenin level in response to *HSPA6* overexpression. **(L)** WB analysis of β-catenin protein expression and phosphorylation in response to *HSPA6* overexpression. *p < 0.05, **p < 0.001, **p < 0.0001.

Since *lnc-TSSK2-8* was mainly distributed in the cytoplasm, we explored whether it could exert its function as a competing endogenous RNA (ceRNA) on DEGs. We predicted that miR-6721-5p targets both the *HSPA6* 3′ UTR and the *lnc-TSSK2-8* full-length sequence (Figure 4A) with the miRDB^2^ (Liu and Wang, 2019; Chen and Wang, 2020). We then validated the interaction between miR-6721-5p and HSPA6-3′UTR/lnc-TSSK2-8 using a dual-luciferase reporter assay. The results suggested that ectopic expression of miR-6721-5p leads to notably reduced luciferase activity of WT *HSPA6* 3′ UTR, while the luciferase activity could not be changed if the binding sites of *HSPA6* 3′UTR were mutated (Figure 4B). Similarly, the interaction of miR-6721-5p and *lnc-TSSK2-8* was also validated (Figure 4C). Overexpression of miR-6721-5p decreased the transcriptional level of *lnc-TSSK2-8* (Figure 4D) but did not affect the gene expression of β-catenin (Figure 4E), while the stabilization of β-catenin was impaired, since its phosphorylation and protein expression were upregulated and downregulated, respectively (Figure 4F). Furthermore, overexpression of *lnc-TSSK2-8* greatly activated the transcription of *HSPA6* (Figure 4G). We overexpressed *HSPA6* to verify its effect on Wnt**/**β-catenin signaling. The result indicated that *HSPA6* (Figures 4H,I) activated transcription of *LEF1* (Figure 4J) and exerted an inverse effect of miR-6721-5p on β-catenin expression (Figures 4K,L).

**FIGURE 5:**
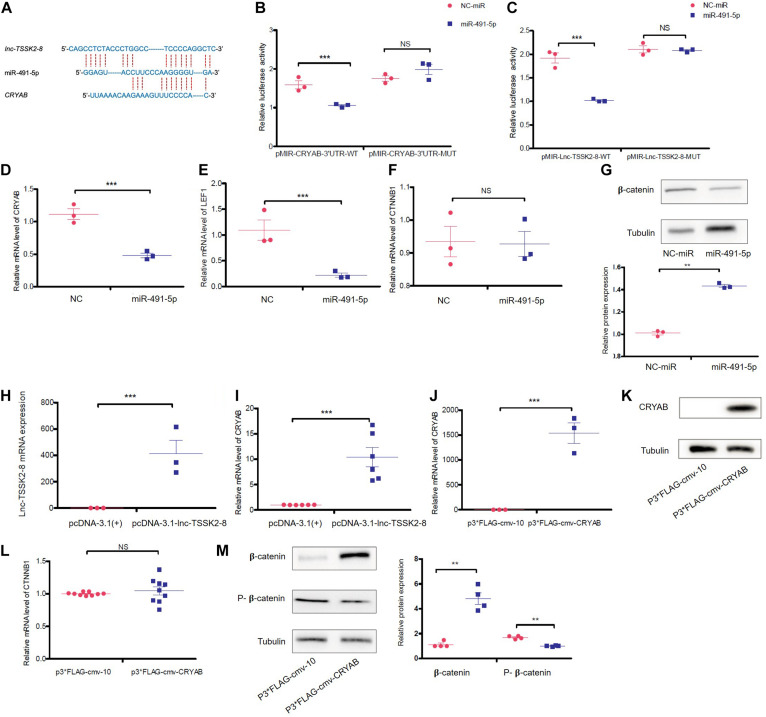
*lnc-TSSK2-8* activation promotes β-catenin stabilization through the miR-491-5p/α-B-crystallin (CRYAB) axis. **(A)** The predicted miR-491-5p binding sites of *lnc-TSSK2-8* and *CRYAB* 3′ UTR. **(B)** The luciferase reporter assay we performed to confirm the interaction between miR-6721-5p and *CRYAB* 3′ UTR **(B)**/*lnc-TSSK2-8* **(C)**. **(D)** RT-qPCR analysis of *CRYAB* level in response to miR-491-5p overexpression. **(E)** RT-qPCR analysis of *lymphoid enhancer-binding factor 1 (LEF1)* level in response to miR-491-5p overexpression. **(F)** RT-qPCR analysis of β-catenin level in response to miR-491-5p overexpression. **(G)** Western blot (WB) analysis of protein expression of β-catenin in response to miR-491-5p overexpression. **(H)** Confirmation of *lnc-TSSK2-8* overexpression with RT-qPCR. **(I)** RT-qPCR analysis of *CRYAB* level in response to *lnc-TSSK2-8* overexpression. **(J)** Confirmation of *CRYAB* overexpression with RT-qPCR. **(K)** Confirmation of CRYAB overexpression with WB. **(L)** RT-qPCR analysis of β-catenin level in response to *CRYAB* overexpression. **(M)** WB analysis of β-catenin protein expression and phosphorylation in response to *CRYAB* overexpression. *p < 0.05, **p < 0.001, **p < 0.0001.

Recently, Mitra et al. (2013) reported that α-B-crystallin (CRYAB) could stabilize β-catenin and promote Wnt signaling in osteogenic differentiation (Zhu et al., 2020). We noticed *lnc-TSSK2-8* and *CRYAB* 3′ UTR also share miRNA target sequences (Figure 5A) and further investigated whether *lnc-TSSK2-8* could regulate Wnt/β-catenin signaling by modulating CRYAB expression. Through dual-luciferase reporter assay, we showed that miR-491-5p could interact with both *CRYAB* and *lnc-TSSK2-8* (Figures 5B,C). Overexpression of miR-491-5p significantly downregulated the transcriptional level of *CRYAB* and *LEF1* (Figures 5D,E) and only inhibits β-catenin expression posttranscriptionally (Figures 5F,G). As expected, overexpression of *lnc-TSSK2-8* greatly increased the transcription of *CRYAB* (Figures 5H,I). At last, we overexpressed CRYAB (Figures 5J,K) and verified its posttranscriptional effect on β-catenin expression (Figures 5L,M).

## Discussion

Wnt proteins are a protein family composed of secreted glycoproteins acting as signaling molecules. They bind to Frizzled and several other coreceptors such as lipoprotein receptor-related protein (LRP)-5/6 to trigger intracellular signal transduction (Logan and Nusse, 2004; Kestler and Kuhl, 2008). In the canonical Wnt/β-catenin signaling pathway, when Wnt signal is absent, β-catenin is phosphorylated by the destruction complex. Such processes include CK1-mediated phosphorylation at Ser45, and GSK3β-mediated phosphorylation at Ser33, Ser37, and Thr41. The phosphorylated β-catenin is degraded by the proteasome through the ubiquitin pathway. When Wnt ligands are available, a cascade initiated by the binding of Wnt to the Frzs is activated. The destruction complex consisting of axin, adenomatous polyposis coli (APC), and GSK3β is disassembled, resulting in the stabilization of β-catenin. The β-catenin molecules subsequently accumulate in the cytoplasm and are imported into the nucleus, where they serve as transcriptional coactivators of the TCF/LEF family transcription factors to regulate gene expression (Rao and Kuhl, 2010). The canonical Wnt/β-catenin signaling pathway has been proven to play important roles in multiple aspects of vertebrate heart development such as cardiac differentiation and right ventricular growth (Ai et al., 2007; Gessert and Kuhl, 2010).

The stability of cytosolic β-catenin is controlled by the destruction complex. As the member of the destruction complex, GSK3β acts as a negative regulatory factor. We found that overexpression of *lnc-TSSK2-8* promoted the phosphorylation of GSK3β. Subsequently, the phosphorylation of β-catenin was decreased, which might inhibit its ubiquitination. We showed that *lnc-TSSK2-8* promotes the stabilization of β-catenin through regulating the expression of *HSPA6* and *CRYAB*. CRYAB is the most abundant small HSP constitutively expressed in cardiomyocytes. It protects against reperfusion injury/myocardial ischemia and suppresses cardiac hypertrophic responses by attenuating nuclear factor of activated T cells (NFAT) signaling (Kumarapeli et al., 2008). CRYAB was also identified as a downstream effector of calcineurin-induced protection against cardiomyocyte apoptosis (Bousette et al., 2010) and a molecular switch in bypassing mitochondrial pathway of apoptosis during myocardial infarction (Mitra et al., 2013). Zhu et al. (2020) recently revealed that CRYAB physically interacts with β-catenin; it promotes osteogenic differentiation of bone marrow stem cells by protecting β-catenin from ubiquitination and degradation (Zhu et al., 2020). HSPA6 has not been reported as associated with Wnt/β-catenin signaling. Our study showed that HSPA6 and CRYAB not only stabilized β-catenin but also activated the downstream transcription factor LEF1. Since dosage of β-catenin significantly affects OFT development upstream of *Tbx1* (Racedo et al., 2017), we propose that the small HSP-mediated stabilization of β-catenin should be a common mechanism involved in heart development. The expression of HSPA6 and CRYAB could be modulated by *lnc-TSSK2-8* through sponging miR-6721-5p and miR-491-5p, respectively. Wnt/β-catenin signaling could potentiate neonatal mouse cardiomyocyte proliferation and human induced pluripotent stem (iPS) cell-derived cardiomyocyte. A Wnt/β-catenin-dependent transcriptional network governing cardiomyocyte proliferation was discovered (Quaife-Ryan et al., 2020). We showed that *lnc-TSSK2-8* promoted cell proliferation of HEK293, which might result from enhancement of Wnt/β-catenin signaling.

Significant achievements have been made in revealing the roles of miRNAs and lncRNAs in heart development (Alexanian and Ounzain, 2020; Das et al., 2020). Cytoplasmic lncRNA could act as ceRNAs to sequester miRNAs from their natural mRNA targets; we established that *lnc-TSSK2-8* regulates *HSPA6* and *CRYAB* in this way. Whereas *lnc-TSSK2-8* also has substantial nuclear expression, the transcriptomic analysis indicated that *lnc-TSSK2-8* might be associated with cilia functions. Cilia have an essential role in the pathogenesis of congenital heart disease (Li et al., 2015; Gabriel et al., 2020). We noted that the DEGs were enriched in dynein binding, which is critical for ciliary processes. The MF of *lnc-TSSK2-8* in cilia process and heart development needs to be further investigated. Conotruncal defects such as TOF, truncus arteriosus, conoventricular ventricle septum defect, type B interruption of the aortic arch account for about 70% of heart malformations associated with 22q11.2DS (Agergaard et al., 2012). Although several genes including *TBX1* have been identified responsible for heart defects resulting from 22q11.2 deletion, noncoding functions of this common CNV in cardiac development have not received enough attention. We demonstrated that the 22q11.2 *lnc-TSSK2-8* is involved in the canonical Wnt signaling pathway by stabilizing β-catenin. There were two main limitations of our study. Firstly, our experiments were based on the HEK293 cell line, which is derived from human embryonic kidney. Additional validation using primary cardiac cells derived from model animals or *in vitro* cardiomyocyte differentiation from human iPS cells would help reveal the cardiac-specific effect of *lnc-TSSK2-8*. Secondly, loss-of-function validation was not performed for *lnc-TSSK2-8*, *HSPA6*, and *CRYAB*. It would be meaningful to carry out si/shRNA-mediated knockdown of *lnc-TSSK2-8*, *HSPA6*, and *CRYAB* to investigate their roles in the context of heart development, which would help elucidate the contribution of *lnc-TSSK2-8* to cardiac phenotypes of 22q11.2DS.

In summary, we established that the 22q11.2 *lncRNA lnc-TSSK2-8* acts as an ceRNA to regulate the expression of HSPA6 and CRYAB through sponging miR-6721-5p and miR-491-5p; subsequently, HSPA6 and CRYAB activates canonical Wnt signaling by promoting the stabilization of β-catenin and activating LEF1 expression. Our study provided evidence of a lncRNA that regulates the dosage of β-catenin, which contributes to the OFT anomalies in 22q11.2DS.

## Data Availability Statement

The datasets presented in this study can be found in online repositories. The names of the repository/repositories and accession number(s) can be found below: https://www.ncbi.nlm.nih.gov/geo/, GSE165927.

## Author Contributions

JF, YG, XpZ, and XqZ performed the lab experiments. BW, MQ, and XyZ performed bioinformatic data analysis. BW wrote the manuscript. QF, YG, ZX, and BW conceived the study and contributed to editing. All authors contributed to the article and approved the submitted version.

## Conflict of Interest

The authors declare that the research was conducted in the absence of any commercial or financial relationships that could be construed as a potential conflict of interest.

## Footnotes

1 https://www.encodeproject.org/

2 http://mirdb.org/
